# Occurrence of multi-oocyte follicles in aquaporin 8-deficient mice

**DOI:** 10.1186/1477-7827-11-88

**Published:** 2013-09-10

**Authors:** Weiheng Su, Xingang Guan, Di Zhang, Meiyan Sun, Longfei Yang, Fei Yi, Feng Hao, Xuechao Feng, Tonghui Ma

**Affiliations:** 1 National Engineering Laboratory for AIDS Vaccine, School of Life Science, Jilin University, Changchun, P.R. China; 2 College of Basic Medical Sciences, Dalian Medical University, Dalian, P.R. China; 3 Central Research Laboratory, Bethune Second Hospital, Jilin University, Changchun, P.R. China

**Keywords:** Aquaporin-8, Multi-oocyte follicle, Folliculogenesis, Formation of follicle

## Abstract

**Background:**

Granulosa cells play a key role in folliculogenesis and female reproduction. Our previous study demonstrated that water channel aquaporin-8 (AQP8) is expressed in mouse follicular granulosa cells and is an important determinant of granulosa cell apoptosis and follicular maturation. More roles of AQP8 in folliculogenesis remain to be determined.

**Findings:**

The present study reports the increased occurrence of multi-oocyte follicles (MOFs) in ovaries of AQP8 knockout mice. The MOFs in AQP8-deficient ovaries contained two or three oocytes, and distributed at various follicle stages including primary (12.5%), secondary (50%), antral (18.8%) and atretic (18.8%) follicles in 5-week ovaries. The MOF is occasionally seen in wild-type ovary only in primary and secondary follicles. The number of MOFs in AQP8-deficient ovary reduced with age (26.7 +/− 5.2 per ovary at 5 weeks old, 14 +/− 5.5 at 10 weeks old, and 3.3 +/− 5.1 at 20 weeks old). mRNA expression of AQP5, AQP7, AQP8, AQP11 and AQP12 was detected in neonatal mouse ovaries and in granulosa cells in 4 week old mouse ovaries. The expression of AQP7, AQP11 and AQP12 mRNAs are decreased significantly in neonatal AQP8-deficient ovaries, whereas AQP5 mRNA expression remains unchanged.

**Conclusions:**

The emergence of MOFs is associated with AQP8 deficiency. The study suggested the involvement of AQP8 in the formation of follicles and provided new insight into the molecular mechanisms of folliculogenesis.

## Findings

### Background

Follicles are functional units of mammalian ovary that develop through primordial, primary, secondary, antral, and preovulatory follicle stages, and finally ovulate in response to the stimulation of luteinizing hormone. Disorders of folliculogenesis in ovary lead to gynecological diseases such as polycystic ovary syndrome, ovarian cyst, premature ovarian failure and even ovarian carcinoma, which impair female reproductive function [1,2]. In some cases abnormal folliculogenesis also can cause increased ovulation [3,4]. However, the mechanisms underlying the altered folliculogenesis are largely unknown.

Aquaporins (AQPs) are a family of water channel proteins selectively expressed on the plasma membrane of some cell types where they may have important physiological functions [5]. Follicles, especially antral follicles are fluid-containing structures. Expression and function of several AQPs in follicular granulosa cells have been reported in human [6], rat [7], mouse [3,8], and pig [9] ovaries. AQPs 7–9 expressed in granulosa cells may provide a transcellular water transport pathway into antrum in the rat follicle [7]. AQP-mediated plasma membrane water permeability can control the rate of apoptosis by regulating apoptotic volume decrease in rat granulosa cells [10]. Thoroddsen et al. reported that AQPs1-4 are differentially expressed in human granulosa and theca cells of the preovulatory follicle during ovulation and may be involved in follicular rupture and corpus luteum formation [11]. Another study by Qu et al. suggested that hyperandrogenism in follicular fluid of women with polycystic ovary syndrome inhibited the expression of AQP9 in granulosa cells through the PI3K pathway [6]. Our previous study identified AQP8 expression in mouse granulosa cells and revealed that AQP8 deficiency increases the number of mature follicles, ovulation and female fertility by reducing the apoptosis of granulosa cells [3]. These studies suggested that AQPs may have important functions in folliculogenesis.

In the present study, we report the increased occurrence of multi-oocyte follicles (MOFs) in the ovaries of AQP8-deficient mice. MOF is also called poly-ovular follicle, which contains more than one oocyte in a follicle and is considered as an anomaly generated from misguided oocyte nest breakdown and primordial follicle assembly, or developing follicle joining [4,12,13]. This new finding further indicates the involvement of AQP8 in the formation of follicles.

### Methods

AQP8-deficient mice in a C57BL/6 genetic background used in this study were described previously [3]. Experiments in this study were performed on age-matched AQP8^−/−^ mice and wild-type mice. Protocols for mouse experiments were approved by the Committee on Animal Research of Jilin University (No. AP12028).

For follicle counting, ovaries were excised and fixed in paraformaldehyde, then embedded in paraffin. Paraffin blocks were serially sectioned at the thickness of 7 μm. The sections were then stained with hematoxylin and eosin (HE). In approximately every 10th ovarian section, the numbers of primary, secondary, antral and atretic follicles with single or multiple oocytes were counted under microscope. The morphological classification of growing follicles was done as described previously [14]. The total numbers of various types of follicle were multiplied by a factor of 10 to get an assessment of the total number of follicles per ovary. Only follicles containing visible oocyte(s) were counted to avoid double counting, and all counting was performed without the knowledge of genotype.

For gene expression analysis, ovaries from neonatal (19.5 days post coitum) or 4-week-old mice were excised, without adipose tissue, capsule and mesovarium. Total RNA of neonatal ovaries or isolated granulosa cells from 4-week-old ovaries was extracted using a TRIzol Reagent (invitrogen, UK). For reverse transcription–PCR (RT-PCR), target genes were amplified from 100 ng total RNA using a SuperScript One-Step RT-PCR kit (Invitrogen, UK), and the PCR products were analyzed by agarose gel electrophoresis. For quantitative RT-PCR (qRT-PCR), target genes were amplified from 100 ng total RNA using a One Step SYBR PrimeScript RT-PCR Kit (TaKaRa, Japan) and CFX96 real-time PCR detection instrument (Bio-Red, USA). Relative gene expression levels were presented by the comparative cycle threshold (ΔΔCt) method [11]. The expression of target gene was normalized to the endogenous control (β-actin) by subtracting the Ct value of the target gene with the Ct value of the endogenous control. To compare levels relative to a calibrator (mean ΔCt for the wild-type group), ΔCt of wild-type were subtracted from the ΔCt of AQP8^−/−^ samples. Relative expression level is given by 2^-ΔΔCt^. The primers used in this study were listed in Table 1.

**Table 1:** PCR primers and product lengths of AQPs and β-actin

**Gene**	**Sense primer**	**Antisense primer**	**Product length**
id-AQP1	5′-caatgacctggctcacggtgt-3′	5′-tctgtgaagtcgctgctgcgt-3′	342 bp
id-AQP2	5′-cgcagtgacaacctgggtag-3′	5′-agagtgcagctccaccgact-3′	327 bp
id-AQP3	5′-gggctgtactacgatgcaatc-3′	5′-acacgaagacaccagcgatgg-3′	421 bp
id-AQP4	5′-tgccagctgtgattccaaacg-3′	5′-gccttcagtgctgtcctctag-3′	469 bp
id-AQP5	5′-tggtcatgaatcggttcagcc-3′	5′-tagggagaggtgctccaaac-3′	300 bp
id-AQP6	5′-cagctgccatgattggaacc-3′	5′-gcaaaggccaagcgtgaatg-3′	374 bp
id-AQP7	5′-tggatgaggcattcgtgactg-3′	5′-cacccaccaccagttgtttc-3′	251 bp
id-AQP8	5′-cttggctaaagtggtgagtcc-3′	5′-agatccaatggaagtcccag-3′	311 bp
id-AQP9	5′-catttgtatccgtgccaggtg-3′	5′-catgatgacgctgagttcgtg-3′	425 bp
id-AQP11	5′-gctctactgcacttccaggag-3′	5′-ctgaacatgaggatcatcatc-3′	227 bp
id-AQP12	5′-cttactacagagcctcatggc-3′	5′-tcttggcgtccacagaacctg-3′	434 bp
id-β-actin	5′-catcctgcgtctggacctg -3′	5′-atctccttctgcatcctgtc -3′	429 bp
q-AQP5	5′-gcatcctgtactggttggcg-3′	5′-aagtagatccccacaagatggc-3′	229 bp
q-AQP7	5′-caacagaactcacagccacc-3′	5′-ggtaattttcacccggcgtc -3′	214 bp
q-AQP11	5′-cacagcgctctactgcacttc-3′	5′-gggttaaacaatgctcctgtgag-3′	119 bp
q-AQP12	5′-ggtcctgtcgcagggatgat-3′	5′-ctttgtgcatcttggcgtcc -3′	154 bp
q-β-actin	5′-ccaccatgtacccaggcatt-3′	5′-ccggactcatcgtactcctg-3′	189 bp

“id” means identification primers used in RT-PCR.

“q” means fluorescent quantification primers used in qRT-PCR.

All primers were designed across introns.

Statistical analysis was performed by SPSS statistics (version 17.0) using two-tailed Student *t* test. Data are expressed as the mean ± SD.

### Results

**Figure 1:**
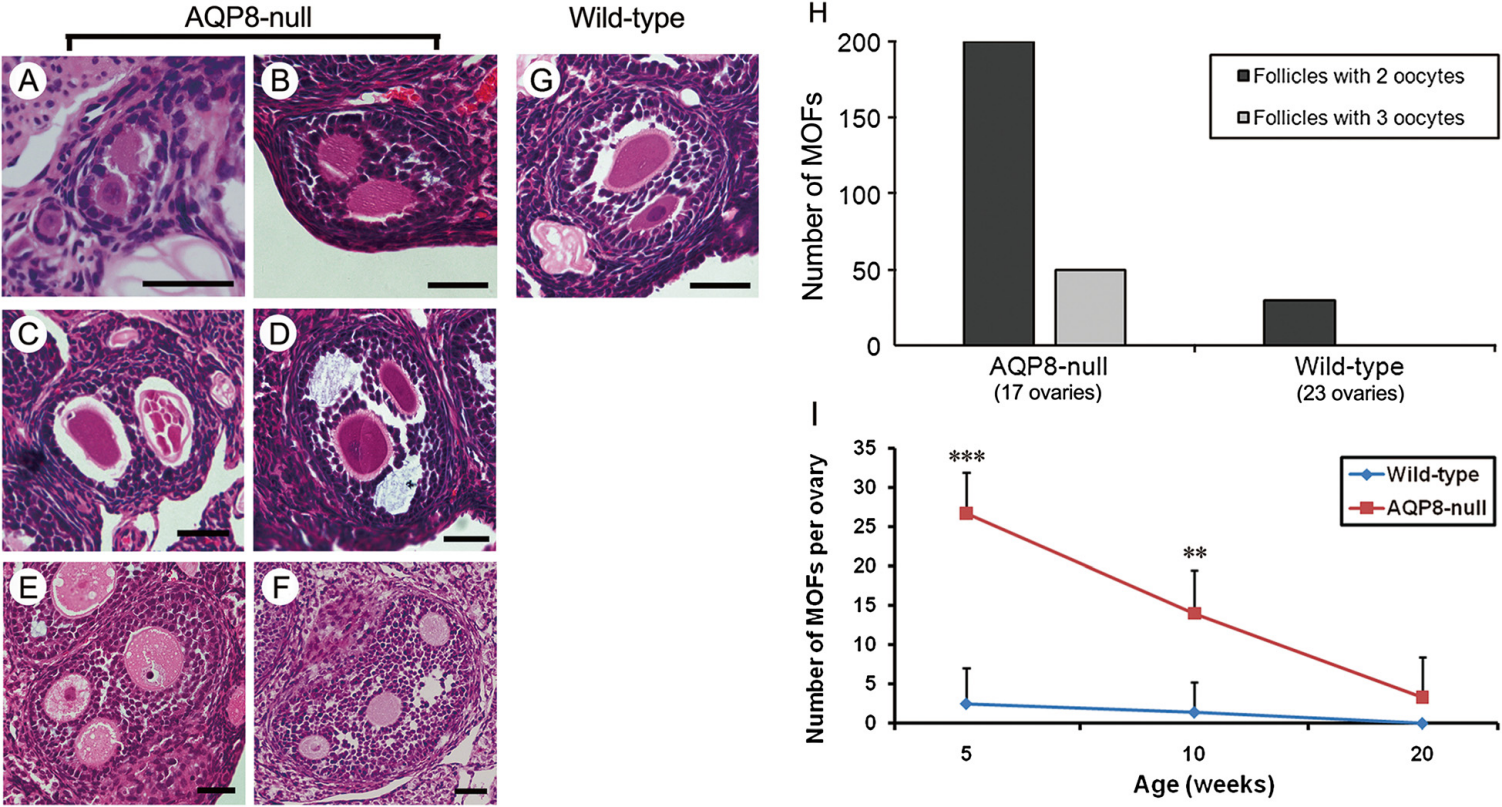
**Morphology and quantity of MOFs in AQP8-deficient ovary. (A-F)**, representative images of MOFs in the HE-staining sections of AQP8 null ovaries. Scale bar, 40 μm. **(G)**, a representative MOF in wild-type ovaries. **(H)**, the numbers of MOF containing 2 or 3 oocytes in AQP8 null and wild-type ovaries. **(I)**, the numbers of MOF per ovary at different ages of both genotypes. n = 5 ~ 8 (shown in Table 2). **: P < 0.01; ***: P < 0.001.

Figure 1 shows the morphology and number of MOFs in the ovaries of AQP8^+/+^ and AQP8^−/−^ mice. We counted 250 MOFs in 17 AQP8^−/−^ ovaries and only 30 MOFs in 23 AQP8^+/+^ ovaries. The MOFs in AQP8^−/−^ ovaries contained two (Figures 1A-D) or three (Figures 1E and F) oocytes. The number of MOFs with two or three oocytes were 200 and 50 respectively in AQP8^−/−^ ovaries, and were 30 and 0 in AQP8^+/+^ ovaries (Figure 1H). According to the classification of follicles, the MOFs in AQP8^−/−^ ovaries were seen in all follicle types including primary follicle (Figure 1A), secondary follicle (Figures 1B and E), antral follicle (Figures 1D and F), and also atretic follicle (Figure 1C). As shown in Figure 1I, ovaries of AQP8^−/−^ mice at age of 5 weeks and 10 weeks presented remarkably increased number of MOF compared with AQP8^+/+^ mice at the same age. The number of MOFs decreases with aging in AQP8^−/−^ ovaries.

The distribution of single-oocyte follicles (SOFs) and MOFs at different follicle stages in AQP8^+/+^ and AQP8^−/−^ mice were summarized in Table 2. MOFs were found in all follicle stages in AQP8^−/−^ ovary, but only in primary and secondary stages in AQP8^+/+^ ovary.

**Table 2:** Distribution of SOFs and MOFs during the follicle development

**Genotype**	**Age weeks**	**Number of ovary**	**Type of follicle**	**Number of follicle per ovary by stages (%)**			
				**Primary**	**Secondary**	**Antral**	**Atretic**
*Wild-type*	5	8	SOF	88.8 ± 20.3 (28.4)	128.8 ± 35.9 (41.2)	31.3 ± 11.6 (10.0)	63.8 ± 26.1 (20.4)
			MOF	1.3 ± 3.5 (50)	1.3 ± 3.5 (50)	-	-
	10	7	SOF	92.9 ± 32.6 (22.7)	138.6 ± 37.2 (33.9)	45.7 ± 22.2 (11.2)	131.4 ± 33.9 (32.2)
			MOF	-	1.4 ± 3.8 (100)	-	-
	20	8	SOF	33.8 ± 18.4 (17.1)	55.0 ± 20. (27.8)1	20.0 ± 9.5 (10.1)	88.75 ± 27.3 (44.9)
			MOF	-	-	-	-
*AQP8-null*	5	6	SOF	85.0 ± 25.8 (25.2)	146.7 ± 44.3 (43.6)	31.7 ± 13.5 (9.4)	73.3 ± 21.4 (21.8)
			MOF	3.3 ± 5.2 (12.5)	13.3 ± 8.2 (50)	5.0 ± 5.5 (18.8)	5.0 ± 5.5 (18.8)
	10	5	SOF	94.0 ± 27.4 (23.6)	170.0 ± 51.7 (42.7)	40.0 ± 14.8 (10.1)	94.0 ± 20.0 (23.6)
			MOF	2.0 ± 4.5 (14.3)	6.0 ± 8.9 (42.9)	2.0 ± 4.5 (14.3)	4.0 ± 5.5 (28.6)
	20	6	SOF	40.0 ± 15.5 (20.9)	66.7 ± 24.1 (34.8)	16.7 ± 7.4 (8.7)	68.3 ± 26.8 (35.7)
			MOF	-	1.7 ± 4.1 (50)	-	1.7 ± 4.1 (50)

*SOF* single-oocyte follicle, *MOF* multi-oocyte follicle.

MOFs are considered as an abnormality which happened during two possible stages: primordial follicle assembly or follicle development. Williams et al. indicated that MOFs are the results of joining between developing follicles [4]. In female T-synthase (core 1 β 1, 3-galactosyltransferase) deficient mice, some adjacent follicles of late development stage showed breached adjoining boundary and confluent contents, and gradually became MOFs. However, we did not see any breached adjoining boundary of follicles in upstream and downstream continuous sections of MOFs in our study, suggesting that most of MOFs in AQP8^−/−^ ovaries are not formed by joining of developing follicles.

**Figure 2:**
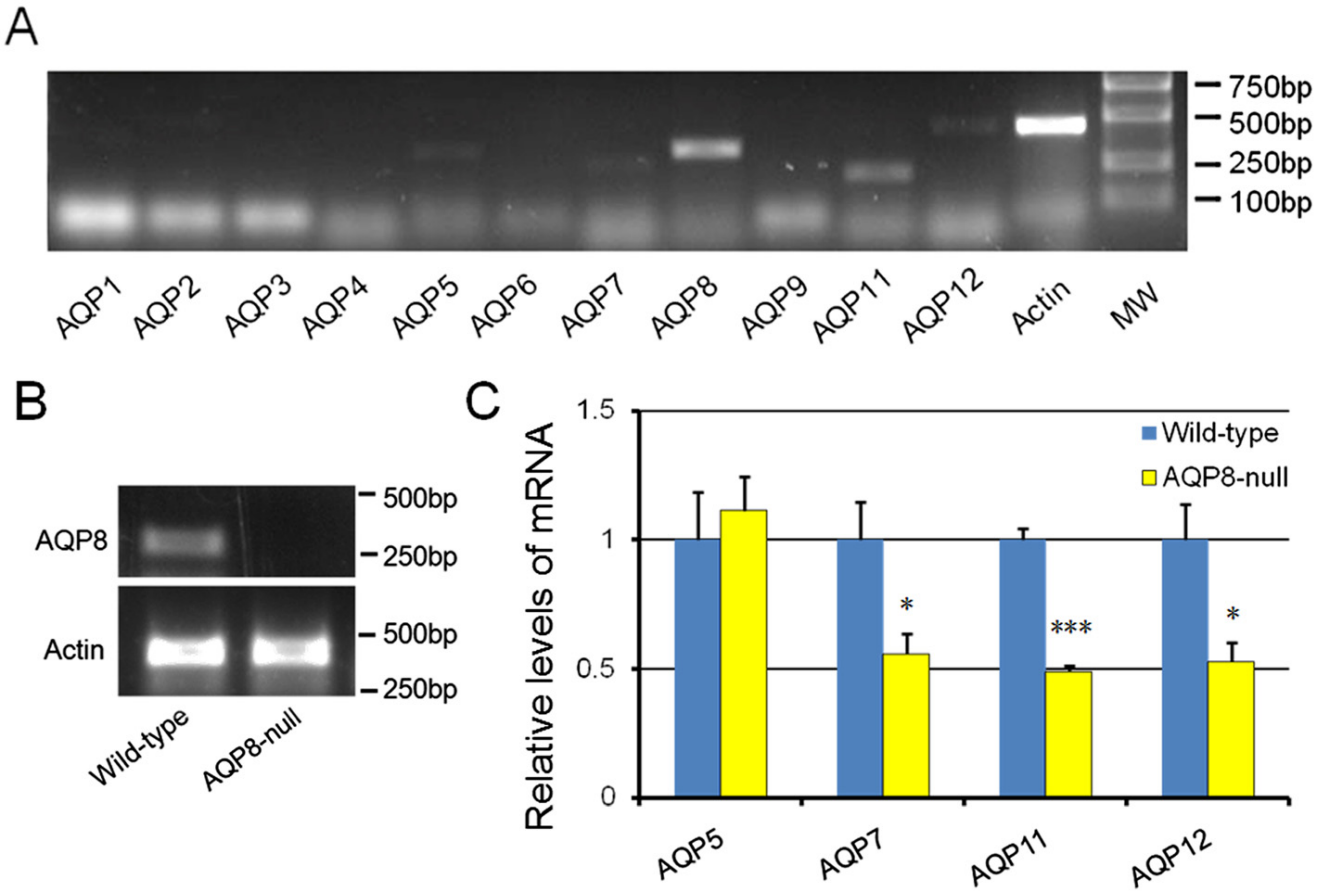
**RT-PCR and qRT-PCR detection of AQP8 and AQPs family expression. (A)**, mRNA expression of AQP family in 4-week-old mouse granulosa cells detected by RT-PCR. **(B)**, mRNA expression of AQP8 in mouse neonatal ovaries detected by RT-PCR. **(C)**, relative mRNA expression levels of AQP5, AQP7, AQP11 and AQP12 in mouse neonatal ovaries detected by qRT-PCR and analyzed by ΔΔCt method. n = 3 separate experiments. *: P < 0.05; ***: P < 0.001 compared with wild-type.

On the other hand in neonatal ovary, the oocyte nests or germ cell cysts breakdown into individual oocytes. Meanwhile, two thirds of the oocytes undergo apoptosis, and the surrounding somatic cells (pre-granulosa cells) invade and encapsulate survived individual oocytes, forming primordial follicles. MOFs are also considered to generate from incomplete or misguided oocyte nest breakdown or impaired differentiation and invading of pre-granulosa cells, resulting in inadequate assembly of follicles [12,13]. This manner probably works for MOFs formation in AQP8-deficient mice. Because oocytes do not express AQP8 [15], MOFs in AQP8^−/−^ mice seem to be induced by altered function of pre-granulosa cells. To analyze the underlying mechanism, the expression of AQP8 and other AQPs in neonatal pre-granulosa cells of both wild-type and AQP8^−/−^ mice were detected. AQP8 was reported to express in granulosa cells of formed follicles, we first investigate the expression of AQPs family in granulosa cells of 4-week-old mice. RT-PCR results indicated that AQP5, AQP7, AQP8, AQP11 and AQP12 are expressed (Figure 2A), and this is the first time to report the expression of AQP5, AQP11 and AQP12 in mouse granulosa cells. For reasons that neonatal pre-granulosa cells are difficult to isolate, and neonatal ovaries are almost simply composed by germ cells and pre-granulosa cells, neonatal ovaries were used to represent pre-granulosa cells in this study. RT-PCR results identified that AQP8 is also expressed in neonatal wild-type ovaries, but not in AQP8^−/−^ ovaries (Figure 2B). Furthermore, relative expression levels of the five AQPs in wild-type and AQP8^−/−^ neonatal ovaries by qRT-PCR indicated significantly decreased AQP7, AQP11 and AQP12 (but not AQP5) expression in AQP8^−/−^ neonatal ovaries compared to wild-type ovaries (Figure 2C).

### Discussion

Molecular mechanisms of MOF formation are not well understood. MOFs were found in mice deficient in BMP15, GDF9, Dmrt4, Ahch, CaMKIV and Akt1 in previous studies [16-20]. These proteins are expressed in ovary and play diverse roles, such as oocyte-secreted growth factors, nuclear hormone receptor, protein kinase and phosphoinositide 3-kinase signaling pathway receptor. Besides, estrogen compounds such as diethylstilbestrol and genistein, and overexpression of *inhibin-α* gene also induced MOFs formation [21-23]. Therefore, the mechanisms underlying the formation of MOFs are complicated and may involve multiple factors and pathways.

In the present study, occurrence of MOFs was found in mice lacking AQP8 that is expressed exclusively in granulosa cells [3] and in neonatal mouse ovary, suggesting that altered granulosa cell or pre-granulosa cell function by AQP8 deletion is responsible for the formation of MOFs. The incomplete penetration suggests that the AQP8 null allele is haploinsufficient or semi dominant for the MOFs phenotype. We didn’t find morphological trace of joining between adjacent follicles, suggesting MOFs are not formed during follicle development but during primordial follicle formation in neonatal ovary. AQP8 is a major plasma membrane water channel of mouse granulosa cells, as AQP8 deletion reduced the water permeability by 45% [3]. Reduced plasma membrane water permeability in AQP8^−/−^ pre-granulosa cells may have two consequences: decreased apoptosis and impaired cell migration [5,10,24] that are likely to change the behavior of pre-granulosa cells during folliculogenesis. Saadoun et al. reported that cell migration of AQP1- or AQP4-transfected cells are faster than that of control cells [24]. In migrating cells which express AQP1, the localization of AQP1 polarized into the lamellipodia, where rapid water fluxes occur. In this context, the migration of pre-granulosa cells is probably impaired in AQP8-deficient mice. Therefore the invasion and encapsulation of pre-granulosa cells may be slowed down by AQP8 deletion, resulting abnormal encapsulation and formation of MOFs. In addition, other factors such as the transport property of AQP8 to urea, ammonia and H_2_O_2_[25-27] and the decreased expression of AQP7, AQP11 and AQP12 may also contribute to the increased formation of MOFs in AQP8^−/−^ mice. So far, there is not report for observation of MOFs in other AQP-deficient mice. Further studies are required to determine the mechanisms of increased MOFs formation in aquaporin deficient ovary.

The MOFs in AQP8^−/−^ ovary happened more frequently in prepubertal mice, decreased with age, and nearly disappeared in old mice. A similar age-related change of MOFs quantity was also observed in dog ovary [28]. Such characteristics of MOFs appearance support their formation in the early stage of folliculogenesis. The distributions of MOF before early antral stage in AQP8-deficient mice are coincident with that of SOF, indicating a similar process of growth and enlargement until early antral stage. However, large antral MOF and preovulatory MOF are rarely seen in ovary sections, suggesting that MOFs may not be able to develop to the late follicle stage. It is unknown whether the increased MOFs contribute to enhanced female fertility in AQP8-deficient mice found previously [3].

### Conclusions

In conclusion, we identified an increased occurrence of MOFs in AQP8-deficient mice. This finding may provide new insights into the molecular mechanisms of follicle formation and regulation.

## Competing interests

The authors have declared that no competing interest exists.

## Authors’ contributions

WS, XG, MS and TM designed the study. DZ, FY and XF carried out the ovary preparations and HE staining. WS, LY and FH counted the data, performed the statistical analysis. WS and MS carried out PCR detection. WS and TM wrote the manuscript. All authors read and approved the final manuscript.
